# Toward a Correlative Metrology Approach on the Same 2D Flake: Graphene Oxide Case Study—Sample Preparation and Stability Issues

**DOI:** 10.3390/nano15241861

**Published:** 2025-12-11

**Authors:** Lydia Chibane, Alexandra Delvallée, Nolwenn Fleurence, Sarah Douri, José Morán-Meza, Christian Ulysse, François Piquemal, Nicolas Feltin, Emmanuel Flahaut

**Affiliations:** 1 Laboratoire National de Métrologie et D’Essais (LNE), 29 Avenue Roger Hennequin, 78190 Trappes, France; lydia.chibane@utoulouse.fr (L.C.); nolwenn.fleurence@lne.fr (N.F.); jose.moran@lne.fr (J.M.-M.); francois.piquemal@lne.fr (F.P.); nicolas.feltin@lne.fr (N.F.); 2 Centre Inter-Universitaire de Recherche et d’Ingénierie des Matériaux Université de Toulouse, Toulouse INP, Centre National de la Recherche Scientifique, 118 Route de Narbonne, 31062 Toulouse, France; 3 Centre de Nanosciences et de Nanotechnologies, CNRS, 10 Bd Thomas Gobert, 91120 Palaiseau, France; christian.ulysse@cnrs.fr

**Keywords:** graphene oxide, correlative metrology, Raman, SPM, SEM

## Abstract

Although graphene promises a wide range of applications, large-scale production of this material remains complex. One very common way of obtaining graphene is through a reduction in graphene oxide (GO). In order to fully control this production process, it is necessary to obtain data from different techniques, but a comprehensive characterization methodology and associated metrology are currently lacking. Here, we propose tools for substrate selection (in this study, the most appropriate were silicon and silicon dioxide on silicon) and precautions to be taken when setting up a correlative metrology method integrating atomic force microscopy (AFM), scanning electron microscopy (SEM), Raman microscopy/spectroscopy, scanning microwave microscopy (SMM) and scanning thermal microscopy (SThM). Indeed, in order to obtain reliable data for each of these techniques applied to a unique graphene oxide flake, a strategy must be developed and could be implemented to monitor the reduction in GO. Emphasis was placed on the choice of the substrate and on the possible degradations generated by each of the techniques employed, and a running sequence was determined.

## 1. Introduction

The development of nanomaterials involves complex processes. The two existing approaches, “top-down” and “bottom-up,” face the same challenges, particularly the control of properties such as size or shape, crystalline structure, and physicochemical stability. In order to meet these challenges and constraints, innovation is needed in characterization and metrology methodologies. An example of a new methodology for graphene oxide (GO) is presented below. This approach is based on the correlative metrology of data obtained from microscopy [1,2,3,4].

Graphene oxide (GO) is a 2D material containing only carbon and oxygen. Almost all oxygen-containing groups are present at the surface and the edges. It has enjoyed considerable popularity in recent years, thanks to its relative ease of production compared to graphene and subsequent numerous proposed utilizations. For example, it could be used in applications such as biosensors [5], composite [6], flexible devices [7], or energy storage [8]. However, the current techniques for producing graphene are often complex and resource demanding. A promising alternative that has progressively emerged is the reduction in graphene oxide.

Graphene oxide reduction leads to reduced graphene oxide (rGO), which is often confused with graphene. During GO reduction, functional groups are eliminated and partial reconjugation of the sp^2^ network of the hexagonal structure occurs. However, this process is not without consequences: the elimination of oxygenated groups can weaken the carbon network, leading to the formation of defects such as holes, vacancies, or the reorganization of atoms, which disrupts the hexagonal structure. In some cases, chemical residues from the reducing agent may also remain, introducing contamination into the material or even an incomplete reduction [9].

These defects affect the mobility of electrons and phonons and increase their dissipation because they modify the density of electronic states and disrupt π delocalization. This leads to variable thermal and electrical conductivities. Moreover, such defects are prime sites for thermal decomposition or catalysts for degradation, lowering the degradation initiation temperature [10]. The degree of reduction and the method and conditions used (type of reducer, temperature, duration, etc.) determine the final defect nature and density and therefore the performance of the rGO.

While suffering from structural defects, rGO is morphologically rather close to graphene, and has similar properties, which opens up even more application possibilities [11,12].

While graphene is an electronic conductor and benefits from good stability in mild conditions, graphene oxide is an insulator and suffers from notorious instability, limiting its development: hence the need for a thorough understanding of its physical and chemical properties and its progressive reduction into rGO.

This study aims to provide tools to implement a correlative metrology approach on graphene oxide (GO) and reduced graphene oxide (rGO) flakes, using the following techniques: Raman microscopy/spectroscopy, atomic force microscopy (AFM), scanning electron microscopy (SEM), scanning microwave microscopy (SMM) and scanning thermal microscopy (SThM).

Each instrument used in this study, taken separately, offers very useful GO physico-chemical characterization, addressing different properties of this nanomaterial. Raman spectroscopy/microscopy is helpful for monitoring the oxidation/reduction process, defect density, sp^3^/sp^2^ ratio, etc. [13,14] and is widely used. AFM and SEM are also very common techniques to visualize the sample or to obtain dimensional and structural data such as lateral measurements for both, and the thickness of flakes for AFM [15,16,17,18,19,20]. Concerning AFM and SEM measurements, standardized methodologies for 2D materials have already been proposed in recent years [19,21]. Although standardized methodologies for SThM and SMM techniques are not yet developed, academic studies show that they can be very helpful to understand thermal and electrical behavior of graphene-related materials, including GO [22,23,24,25,26,27,28,29,30]. However, for a comprehensive investigation of this promising 2D material, it is crucial to understand the relationship between the physico-chemical characteristics. The correlative approach, combining several characterization techniques, is designed to address this issue, especially because different flakes in the same sample may have rather different size, chemical composition, or thickness, and bulk measurements are thus not appropriate to accurately describe the situation.

However, the data acquired that are available in the literature are rarely correlated on a single flake. Many studies show the use of more than one technique [31,32,33,34,35], but not on exactly the same flake or group of flakes. Therefore, the correlation of data from both (or more) techniques is in most cases subject to sample inhomogeneity. This is particularly disadvantageous when using local microscopy techniques, which are often time consuming.

**Figure 1:**
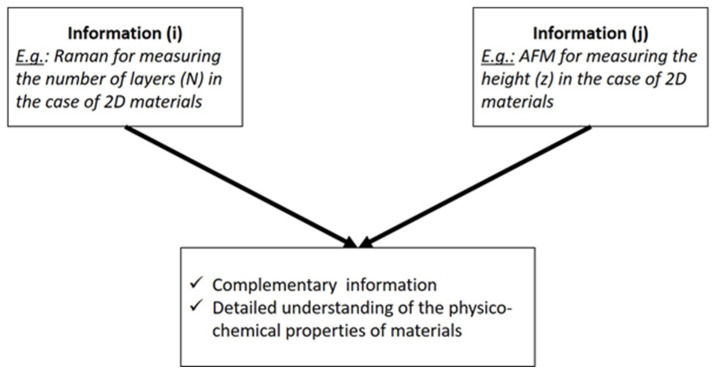
Diagram explaining the concept of correlative metrology.

Although the solution of studying exactly the same object quickly springs to mind, implementation is less straightforward. Moreover, the methodology and necessary care to be taken to implement such a correlative characterization are lacking in the literature. This work aims at dealing with these challenges when implementing a correlative metrology process. Correlative metrology consists of measuring the same object using several techniques, providing different quantities in order to identify correlations between different properties (Figure 1). These different quantities include various physical or chemical information.

However, in order to implement this methodology, several steps have to be established. First of all, the choice of a substrate is a crucial one for any observation of a sample by microscopy, regardless of the technique used. Some techniques require the substrate to have a very low roughness (AFM [36]), some others to be electrically conductive (conductive AFM [37,38] and derived methods such as SMM [24,25], SEM [39], or single-entity electrochemistry, SEE [40]), or need particular optical properties (such as Raman [41]).

The correlative metrology approach has specific requirements. Firstly, the substrate must be suitable for all the techniques that the microscopist wants to employ. If a technique needs a specific condition and another needs the opposite of this requirement, two cases should be considered. The worst case is to be resigned to remove a technique from the study. The alternative case, which is not always possible, is to make a compromise between the opposite needs, considering that this will degrade the results for both techniques.

Moreover, a key issue of correlative microscopy is accurate spatial localization, to ensure that the same region of interest (ROI) may be observed and analyzed, i.e., colocalized, with the different techniques. Some systems exist to combine two microscopy techniques [42,43,44] within the same instrument, but this ideal situation is costly and often restricted to two given techniques. Another option is to localize a ROI from a coordinate system of a first technique and to use a device [45,46,47] to transfer it to another coordinate system of the other technique. The device must thus be attached to the sample. Finally, the solution used in this study is to directly mark the substrate onto which the sample is deposited, and to transfer the substrate between the different microscopy techniques, without touching the sample itself [48].

Moreover, as all the data are not acquired simultaneously, precautions must be taken to ensure that the observation with one technique does not damage the sample before the following observation.

This work addresses the choice of an appropriate substrate and the description of a methodology for the following techniques employed in a correlative approach for the investigation of graphene oxide: AFM, SEM, Raman, SThM, and SMM. It should be noted that the goal of this work is to highlight a methodology rather than perform a purely physical and chemical characterization of the test material.

## 2. Material and Methods

### 2.1. Equipment

All the equipment used for the characterization is available at LNE (Laboratoire National de métrologie et d’Essais, Trappes, France), on two platforms developed for nanometrology. SEM, AFM, SThM, and Raman are located on the CARMEN (Caractérisation métrologique des nanomatériaux) platform dedicated to dimensional, structural, thermal, and chemical properties of nanomaterials and SMM belongs to the NAEL (Nanométrologie électrique) platform, devoted to electrical metrology at the nanoscale.

The scanning electron microscope (SEM) used in this study is a FEG Zeiss Ultra Plus (Zeiss, Oberkochen, Germany). It has been metrologically qualified [49]. This SEM is placed on a massive concrete block to avoid any problematic noise.

The atomic force microscope dedicated to dimensional measurement is a Dimension 3100 with a Nanoscope V controller (Bruker, Palaiseau, France). It has also been metrologically qualified [36]. This AFM is placed on a massive concrete block and enclosed in an acoustic protection box to avoid any noise issues.

The Raman system is a Labram SOLEIL provided by Horiba (Loos, France). It is equipped with two lasers (532 nm and 785 nm) and two gratings (600 grooves, 1800 grooves). The Raman system is placed on an anti-vibration table.

NX-Hivac (from Park Systems Europe, Orsay, France) AFM, available on the MATIS (MATériaux pour l’Industrie et la Société) platform, is used as a scanning thermal microscope (SThM) with Paladium (Pd) resistive probes called KNT probes (manufactured by Kelvin Nanotechnology, Glasgow, UK). This SThM is enclosed in a specific enclosure that can be used to operate under a controlled atmosphere.

The scanning microwave microscope (SMM) consists of an Agilent 5600LS AFM interfaced with a vector network analyzer (VNA) P9374A (both from Keysight technologies, Santa Rosa, CA, USA). The VNA generates a continuous microwave signal with a typical frequency up to 20 GHz, which is delivered via the AFM tip to the sample. Depending on the electrical properties of the sample, part of the microwave signal is reflected. The ratio between the reflected and incident microwave signals and the reflection coefficient *S_11m_* or scattering parameter, is then measured by the VNA [50]. The SPM is placed on an active antivibration table inside a glove box (MBraun) filled with nitrogen gas. The whole set-up (glove box and VNA) is installed in a shielded room equipped with a controlled air conditioning system.

The reduction process was carried at CIRIMAT (Centre interuniversitaire de recherche et d’ingénierie des matériaux) in CNRS—Toulouse University. We used the tubular furnace (ERALY, Fontenay le Fleury, France), which was capable of reaching a maximum temperature of 1100 °C. The furnace is equipped with a 1.5 kW heating element.

### 2.2. Materials

#### 2.2.1. Graphene Oxide and Reduced Graphene Oxide

The graphene oxide used in this work is a powder provided by Graphenea (San Sebastian, Spain, batch reference GOP21007). A suspension was prepared at a concentration of 500 mg.L^−1^ in milli-Q water. This suspension was then diluted 20 times in order to obtain a suitable concentration for further deposition. This suspension was visually stable but re-homogenized in an ultrasonic bath (Branson, MO, USA, 150 W, 20 min) before each deposition.

Thermal reduction in graphene oxide (GO) deposited on a silicon substrate was carried out in the tubular furnace. Before treatment, the system was purged with argon to remove residual air, then placed under a continuous flow of hydrogen (5 L.h^−1^). The sample was then heated to 800 °C at a rate of 600 °C h^−1^ and held at this temperature for 1 h. After treatment, the furnace was ventilated under an inert atmosphere until it returned to room temperature.

#### 2.2.2. Substrates

The choice of substrate was a crucial step before characterization started. Specifications were therefore drawn up, guided by the specific requirements of all the techniques. These specifications imposed several criteria: low surface roughness so as not to interfere with dimensional analysis, good thermal and electrical conductivity to allow for charges to be dissipated (SEM) and prevent overheating (Raman laser), and no interference between the substrate and GO properties.

For example, despite its good electrical conductivity, smooth surface, and chemical compatibility with GO, HOPG was ruled out. Indeed, its crystalline structure, composed of C atoms, is very similar to GO, which would cause interference during the Raman and SEM-EDS analyses. On the other hand, mica would be the perfect substrate for AFM measurements due to its exceptionally smooth surface and its hydrophilic surface, which allows for good initial adhesion of the deposited materials. However, it is highly insulating electrically (charge accumulation in SEM) and thermally (excessively high temperatures under the Raman laser beam). In order to compensate for the lack of thermal and electrical conductivity, the use of metallic substrates as gold may be considered. However, this type of substrate has two drawbacks for our application: it is very rough over large areas, it is expensive, and it is difficult to lithograph.

It was therefore necessary to find a compromise and to choose a substrate that would work in all instruments, even though it was not perfect in every aspect.

Two kinds of substrates were used to implement correlative metrology: silicon (Si) and silica on silicon (SiO_2_/Si). As discussed previously, the chosen substrates are a good compromise between the requirements for each technique used in this work. They are suitable for all the techniques in terms of roughness, and a compromise is found for electrical and thermal conductivity. SiO_2_/Si was also chosen for its optical properties: it is possible to see graphene with an optical microscope if the thickness of the SiO_2_ layer is about 285 nm [51]. The signal is also amplified in Raman spectroscopy [41]. This silicon dioxide layer was realized by Neyco (Vanves, France).

**Figure 2:**
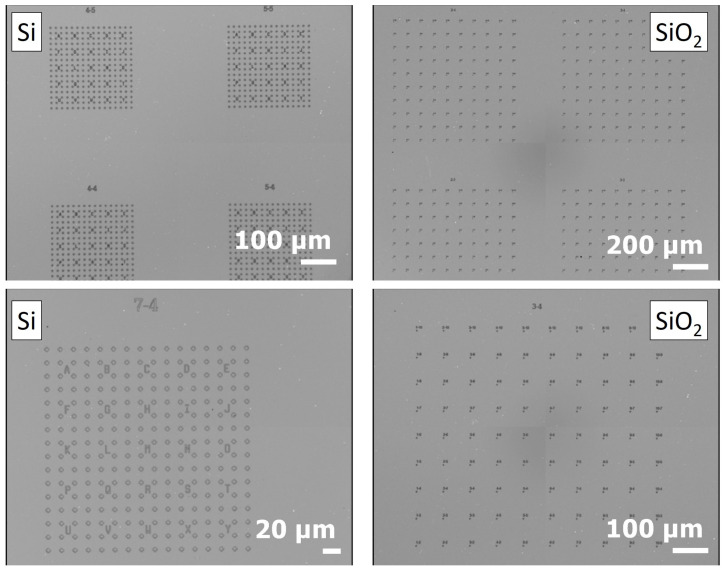
Optical microscopy image of silicon (Si) and silicon with a silica (SiO_2_) layer (285 nm) marked substrates.

Lithography was used with both types of substrates to create marks corresponding to crosses, letters, and numbers. The tracking system was specifically designed by LNE and C2N (Center for Nanosciences and Nanotechnologies, CNRS) and fabricated by C2N. For silicon substrates, the details may be found in reference [48]. A similar design was realized for the SiO_2_/Si substrate. The fabrication of these samples was conducted on 2 inch diameter silicon wafers coated with a 285 nm thick layer of wet thermal oxide (silicon dioxide, SiO_2_). The patterns were created using a combination of electron beam lithography and dry etching. A Raith-Vistec EBPG 5200 electron-beam lithography system was used to define the patterns in a PMMA (polymethyl methacrylate) positive resist. After the development step, which involved immersing the PMMA in a 1:3 MIBK/IPA solution, the mask was transferred using a reactive ion etching (RIE) process with SF6 and CHF_3_ gases. Finally, the silicon wafer was cut using a DISCO DAD 641 dicing saw. An observation of both kinds of substrates with an optical microscope is shown in Figure 2:

#### 2.2.3. Deposition Method

In order to avoid agglomeration of the GO flakes during the deposition process, LNE developed a methodology using poly-L-Lysine (PLL) and a spin-coater. PLL is a cationic polymer which facilitates the adhesion of GO onto the substrate. The substrate was dipped in a PLL solution for 1 h at 4 °C. Then, the spin-coater was used, following a two-step protocol:

The first step consists of spreading a 7.5 µL drop of the GO suspension onto the substrate with a slow spin speed (1000 rpm) for 60 s. This speed and duration are a good compromise and allow for the deposition of isolated flakes onto the substrate.

The second step is dedicated to the very quick (10 s) drying of the drop at 8000 rpm. The liquid (here, water) is eliminated.

**Figure 3:**
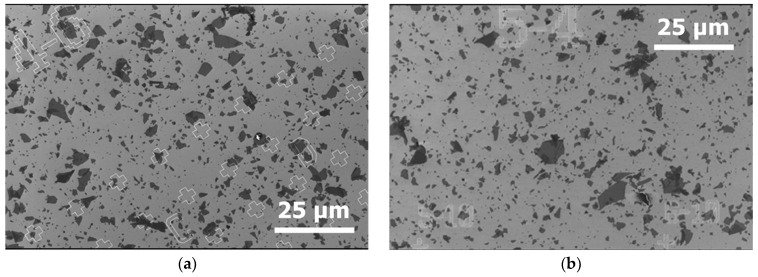
SEM image of GO, deposited with the “spin-coating” method on (**a**) silicon substrate and (**b**) silicon substrate + 285 nm of silica layer.

Figure 3 shows SEM images of the deposits obtained for both types of substrates.

For both types of substrates, the SEM observations are similar. Some overlapping of flakes is observed, but this is not the majority of cases. All the substrates are covered by the flakes with a similar coverage rate.

## 3. Results and Discussion

In this section, we will discuss the damage that each technique employed in this study could bring to the sample, i.e., AFM, SEM, Raman, SMM, and SThM (discussed together in the rest of this paper). A ranking of the damaging level will be shown in order to determine a running sequence of the techniques.

The selected techniques aim to establish a comprehensive overview of the dimensional, structural, thermal, and electrical properties of the samples. It should be noted that certain methods, such as SMM, do not allow for a good electrical contrast in the case of GO due to its insulating nature. We chose to retain this technique in order to assess the type of damage it can cause and to obtain data on the initial product, even though it does not provide precise electrical characteristics for our GO.

However, this technique is being optimized in order to monitor the evolution of GO during its reduction. Indeed, as the material is reduced, its electrical conductivity increases, gradually allowing for an electrical contrast to appear on the SMM images.

### 3.1. Methodology

**Figure 4:**
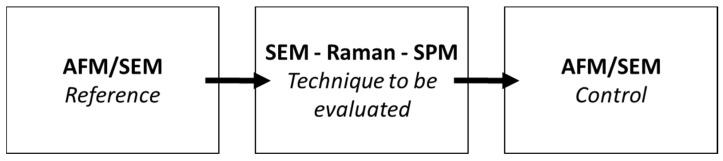
Diagram of the methodology used to determine the level of degradation by technique.

The first thing was to determine what kind of degradation could be observed using the different techniques employed in this study, i.e., SEM, Raman, SThM, and SMM. The last two will be evaluated together and identified as “SPM techniques in contact mode”. A diagram of the methodology used is presented in Figure 4.

The first reference point of the GO flakes deposited onto the substrate must first be realized. This control point may be obtained with AFM in tapping mode or SEM, as these techniques are largely considered to be non-destructive. However, although SEM does not directly affect the object being characterized, this technique can contaminate the sample surface. Studies have shown that amorphous carbon deposition is possible if precautions are not taken [49,52,53,54]. This is why, as with the other two techniques, the state of degradation following its use was also assessed. Thus, SEM, Raman, and SPM will be realized on exactly the same flake image, on the reference point. Lastly, a control of the flake will be made in order to observe any damage.

### 3.2. Observed Damages for Each Characterization Technique

#### 3.2.1. Degradation by SEM

In this case, the AFM in tapping mode was used as the reference technique. We have chosen to work on GO flakes deposited on silicon substrate to avoid possible charge effects in the case of the SiO_2_/Si substrate.

**Figure 5:**
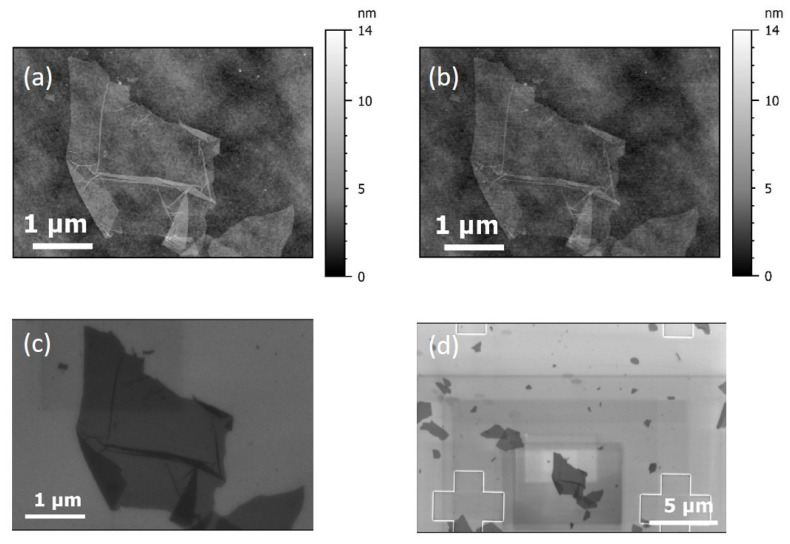
(**a**) AFM reference image of the ROI before electron beam irradiation, (**b**) AFM image of the ROI after electron beam irradiation, (**c**) SEM micrograph of the ROI, and (**d**) larger SEM observation of the ROI after successive zoom out.

A ROI was identified and observed by AFM before and after the electron irradiation with SEM. AFM images with scan size 5 µm × 5 µm, 1024 × 1024 pixels, scan speed of 4 µm·s^−1^ were recorded. OTESPA-R3 tips were used (provider Bruker). SEM image parameters are listed below: the landing energy was set to 3 kV and the working distance to 4.7 mm. An in-lens detector was used. In fact, this detector senses the secondary electrons emitted close to the surface, making it particularly sensitive to surface topography. In order to limit carbon contamination, the sample was left into the SEM chamber in vacuum one night before imaging [49]. The results are gathered in Figure 5.

On the AFM reference image of the ROI (before irradiation), some flakes and folds are observed. Thanks to the localization system, the ROI was easily found with SEM. Two SEM images with different magnifications are shown on Figure 5: the image (b) was acquired at a magnification of 15k× and the second SEM image (c) was acquired by zooming out to a magnification of 5k×. On the zoom out, Figure 5c, marks on the SEM image are clearly visible. They correspond to the amorphous carbon deposition during SEM electron beam irradiation, even if precautions were taken. It is worth raising the question of possible modifications/degradations of the substrate surface state. A control AFM image was obtained after SEM imaging. The comparison between the roughness parameters outside the flakes on the images before and after SEM revealed that they were strictly comparable to *S_q_* = 0.7 nm (root mean square roughness), even though contamination marks are visible by SEM.

SEM, offering both speed and a wide field of view, is the technique of choice for visualizing surface conditions. Before using it as a control method, we made the necessary corrections and improvements to optimize its performance and to be sure not to affect the sample. Specifically, the pumping time was extended, adjustments were made outside the ROI, and low magnifications were preferred to ensure effective and clean analysis.

#### 3.2.2. Degradation by Raman Analysis

The methodology was applied on GO deposited on the SiO_2_/Si substrate for the Raman mapping study. SEM imaging of the ROI was carried out before and after Raman mapping.

The Raman mapping was realized with the 532 nm laser and ×100 objective. The pixel size was 1 µm and the irradiation time by pixel was about 20 s. The power was set to 0.74 mW. This power is adapted to obtain a good signal-to-noise ratio for this kind of substrate. The objective was to obtain spectra with an acceptable signal-to-noise ratio (S/N). We set a threshold of S/N > 20 to consider a good quality spectrum.

**Figure 6:**
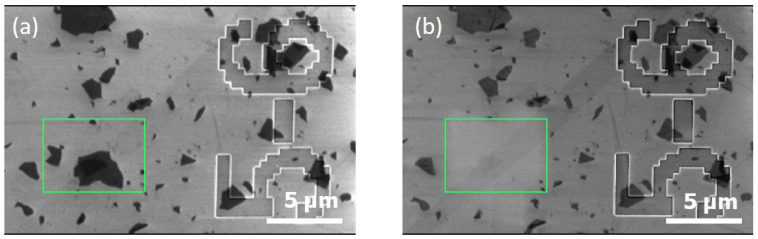
(**a**) SEM reference image of the ROI before and (**b**) after laser irradiation during Raman analysis of GO deposited on SiO_2_/Si substrate. The green boxes indicate the location of the ROI.

The disappearance of the flakes observed in the ROI after Raman mapping is evidenced in Figure 6. The flakes in the surroundings of the ROI were still present and did not seem to be damaged. One explanation could be the local heating of the silica surface. In fact, the thermal conductivity, ***k_SiO2_***, of bulk-fused silica is relatively low (1.28 W·m^−1^·K^−1^ at room temperature [55]), meaning that, inside the substrate, the heat propagation of the local heat flux density induced by the laser impact is quite low. As a result, a high thermal gradient appears between the top and the back surface of the sample, with a high temperature increase at the irradiated surface. Moreover, the GO is particularly unstable with respect to thermal heating. This phenomenon is well known for GO and is used to reduce it very locally [56].

The same methodology was applied to GO deposited on the silicon substrate. Here, AFM was used as a reference for imaging before and after laser irradiation. The Raman mapping was carried out with the 532 nm laser and ×100 objective. The pixel size was 1 µm and the irradiation time by pixel was about 20 s. The power was set to 18 mW. This power is adapted to obtain a good signal-to-noise ratio for this kind of substrate.

**Figure 7:**
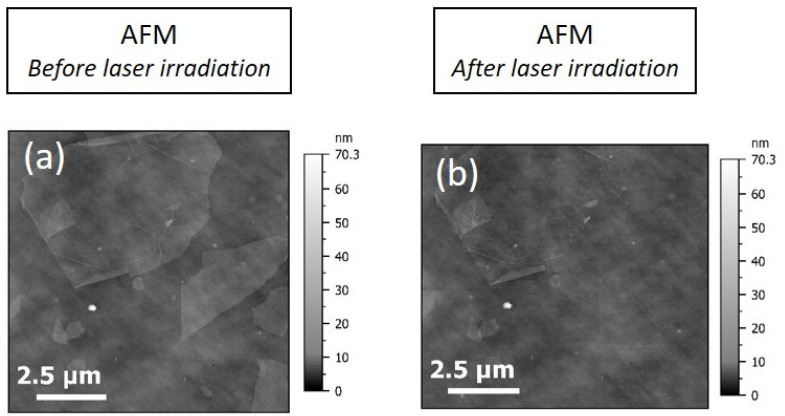
(**a**) AFM reference image of the ROI before laser irradiation, (**b**) AFM image of the ROI after laser irradiation of GO deposited on Si substrate.

Figure 7a indicates that before irradiation, GO flakes were clearly observable within the ROI. After laser irradiation (Figure 7b), an AFM image was acquired from exactly the same ROI. The GO flakes on the left at the bottom almost disappeared. The flakes in the neighborhood of the ROI were still present, showing that only the targeted flake or those in a close neighborhood were affected by the laser.

**Figure 8:**
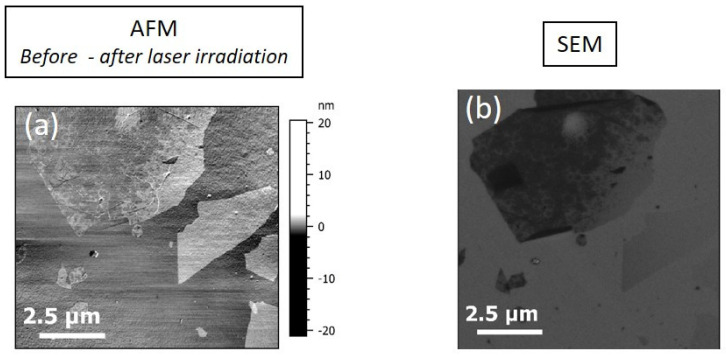
(**a**) AFM reference image of the ROI, subtracted from AFM image after irradiation and (**b**) SEM image of the ROI.

In order to go further in the analysis of the generated data, the AFM image before the irradiation was subtracted from the AFM image after irradiation. This operation facilitates the visualization of the boundaries of flakes, which is difficult to discern by AFM due to the roughness of the substrate, and highlights damage to the flakes. An SEM image was also acquired, as seen in Figure 8.

On Figure 8a, positive values show the degradation of the flakes (or part of the flakes). It reveals that particularly the flakes on the bottom right have suffered damages. Moreover, on the upper right side of Figure 8a, the roughness seems to have increased. The percentage of the area affected by laser irradiation from Figure 8a had been evaluated and the result is ca. 15%. Nevertheless, the observations on the SEM image in Figure 8b still show the presence of one of the flakes, albeit with much less contrast than those that are less damaged. It must also be noted that the flakes are less impacted on silicon, even if the laser power is much higher than the one used on the SiO_2_/Si substrate. In fact, at room temperature, the thermal conductivity ***k_Si_*** of bulk silicon (148 W·m^−1^·K^−1^) [57] is over a hundred times higher than ***k_SiO2_***, which leads to greater heat propagation and limits the temperature rise at the surface of the sample.

To conclude, Raman spectroscopy/microscopy appeared to be a very invasive tool to characterize graphene oxide, certainly leading to very localized local thermal reduction. Following Raman measurements, if the same flake has to be inspected further with another microscopy technique, some precaution must be taken to avoid damage and even more disappearance of the flake. In addition, the interaction with the laser can modify the oxidation state of the sample, which may compromise the integrity of the results. The optimization of settings for that purpose includes at least exposure time to a laser, and laser power. Moreover, in order to preserve the quality of the flake, it is recommended to enlarge the pixel size and to work at the lowest possible magnification. It is advisable to adjust the setting parameters outside the ROI, on similar flakes.

#### 3.2.3. Degradation with SPM (SMM and SThM) in Contact Mode

We have selected the silicon substrate to carry out the correlative process with SMM on GO flakes, where the complex reflection coefficient |*S*_11,m_| is measured [50]. The numerical value of |*S*_11,m_|, the magnitude of *S*_11,m_, is measured in dB. The maximum value of |*S*_11,m_| is 0 dB if 100% of the wave is reflected from the sample. The minimum *S*_11,m_ is achieved when the full wave is fully absorbed by the load.

**Figure 9:**
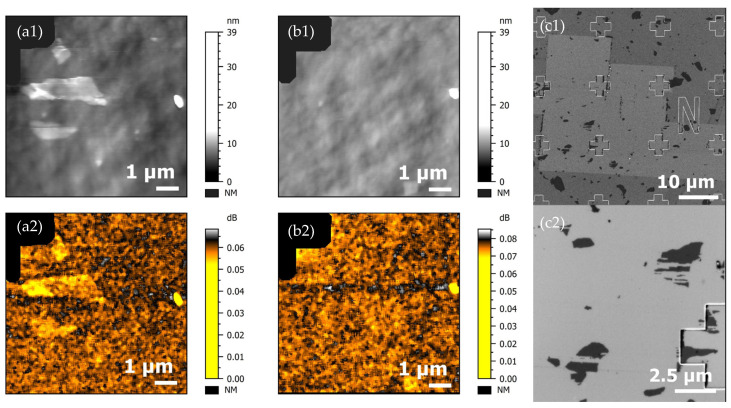
(**a1**,**a2**) Topography and S_11m_ magnitude image (1st acquired scan) of GO 2D flakes on silicon substrate, (**b1**,**b2**) topography and S_11m_ magnitude image (2nd scan)—NM corresponds to the excluded point from the data processing (to facilitate the Z scale reading), (**c1**) large field SEM image of the ROI, and (**c2**) SEM image of the surroundings of the ROI.

It was not possible to acquire an AFM/SEM image of a ROI before acquisition: the optical microscope of the SMM system (Keysight 5600 LS) has a low lateral resolution, making a perfect relocation impossible. To obtain an idea of the possible degradation of the GO flake, the SMM scanning was performed two times on the ROI, using a 25 Pt 300 A SMM probe (from Rocky Mountains Nanotechnology) with a contact force of about 2.2 µN. As a result, the *S*_11m_ magnitude was acquired and revealed small variations of *S*_11m_ signals when the SMM tip scanned different materials: here, GO and Si. A SEM control was carried out after SMM measurements. Figure 9 shows the evolution of the GO flake along the correlation approach.

Three GO flakes were observed on the first SMM scan in contact mode (Figure 9(a1,a2)). A small variation in the *S*_11m_ magnitude was measured between GO flakes and the silicon substrate, in exactly the position of the flakes identified in the topography channel. Moreover, instabilities of the tip (jump to contact) were observed during the first scan. On the second scan (Figure 9(b1,b2)), the GO flakes were removed and no electrical contrast on the ROI could be identified. A large field SEM control image (Figure 9(c1,c2)) of the ROI shows that the area had been “cleaned” from a contamination layer. Here, the pile stayed on the tip during the scan but were ejected on the edge of the fast scan line. Moreover, flakes were also removed. The surroundings, where tests of the apply forces (1 µN) were achieved, were also “cleaned”. Some research teams proposed a protocol based on this principle to clean graphene [58]. Figure 9(c2) shows an area near the ROI where set-up tests were performed: GO flakes are still present, but damaged (scratched).

A recommendation in this case (contact mode) is to control the force applied to the tip as much as possible. Since very specific probes are used for these SPM modes in contact mode, it is not always possible to select a tip that is perfectly suited to the application, particularly in terms of the cantilever’s stiffness. This point is critical in the case of the correlation process.

The same process was applied with the SThM technique. The reference image was acquired in tapping mode with a Bruker Dimension 3100. The scan size of the ROI was set to 5 µm × 5 µm. Then, a thermal contrast mapping was performed with the Nx Hivac SThM on the ROI. Assuming that the thermal contact resistance between the tip and the sample is constant, the thermal image obtained is representative of the thermal conductivity/conductance contrast existing in the sample. This measurement is realized in contact and active mode using a resistive electro thermal probe. The thermal probe is self-heated (by the Joule effect) and acts both as a sensor and as a heater. The heat lost from the hot tip in contact with the cooler sample surface depends on the thermal conductivity of the sample, amongst other parameters. The higher the thermal conductivity of the sample, the greater the amount of heat transferred to the sample, and the more the probe cools down.

**Figure 10:**
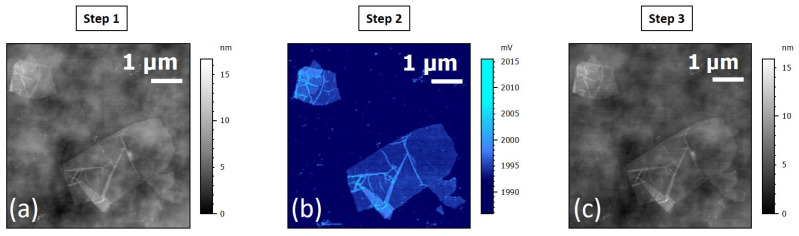
(**a**) Reference topography image in AFM tapping mode. (**b**) SThM data: thermal contrast in contact mode, respectively. (**c**) AFM image of the ROI after SThM acquisition.

When the probe cools down, its electrical resistance decreases. This change in the electrical resistance value is measured using a Wheatstone bridge and results in a change in SThM error value of the Wheatstone bridge. The specific probe used here is a resistive Pd probe. Such probes are quite expensive and fragile (high sensitivity to electrostatic shocks) and supply difficulties are being experienced. They require specific care when handling and using them. We recommend limiting the probe’s current supply level to a maximum of 1 mA. After SThM observation, a control was performed with AFM in tapping mode, with *t* scan sizes: 5 µm × 5 µm. Moreover, a SEM control image was acquired in the end. The results are shown in Figure 10.

The AFM reference image (Figure 10a) of the ROI shows a GO flake with many wrinkles and probably some folds. Few contaminations are visible on the substrate. Figure 10b shows the acquired data with the SThM instrument: thermal contrast was acquired in contact mode.

On the thermal contrast image, the whole flake and wrinkles are distinguished from the rest of the image, which confirms that the thermal conductance of GO is lower than the conductance of the Si substrate (148 W.m^−1^.K^−1^). The control image (Figure 10c) confirms the absence of additional contaminations after scanning. This means that the applied force was adapted to the substrate, although this remains a very important parameter to control during contact mode analyses. The other setting to control to avoid any impact on the sample is the probe’s current.

Another point of discussion concerns the possible reduction in GO due to the tip heating. During SThM measurement in ambient air, on silicon and out of contact, at room temperature, the literature shows that the temperature at the apex is less than 100 °C for the current and the tip used in this study (0.9 mA) [59,60]. At this temperature, we assume that no reduction can be observed on GO.

### 3.3. Suggested Running Sequence by Technique

Tapping mode AFM is perfectly suitable for the observation and measurement of all dimensions of GO flakes, including their thickness. This technique, which is well known to avoid the degradation of the samples and tip [61], perfectly fulfills this statement and respects flake integrity. However, it is perceived as being slow and limited in the size of the scan. This drawback is the same for SPM contact modes such as SThM and SMM. In addition, these techniques, precisely because they are contact techniques, may damage the graphene oxide by removing it with specific probes and generate contamination that disturbs the measurement.

On the other hand, SEM is a fast, easy-to-implement technique to obtain dimensional information (limited to lateral dimensions). However, in a correlative approach, the observed carbon contamination can disturb both the dimensional and chemical information acquired, following SEM imaging.

Finally, Raman spectroscopy/microscopy technique is one of the most employed techniques for graphene oxide characterization and can give chemical/structural information. Unfortunately, if it is not well controlled, damages could be irreversible and may even lead to the disappearance of the GO flakes under investigation.

For all these reasons, we suggest a running sequence (Table 1) for the investigation of graphene oxide through correlative metrology.

Indeed, the main constraint of SPM techniques in contact mode is the force applied by the tip, which was minimized to reduce the stress on the sample as much as possible. The electron beam, combined with an insufficient vacuum level in the SEM, creates an amorphous carbon deposit on the substrate. This issue was mitigated by improving the vacuum level as much as possible and by reducing the magnification. Finally, yet importantly, the Raman parameters were adjusted to limit the impact of the laser beam (local heating) on the material. While satisfying the S/N > 20 condition, both laser power and exposure time have been reduced to a minimum (7 mW), as well as a lower resolution (50×). This power, combined with a higher amount of accumulation (integrations) is a good compromise to obtain a good S/N ratio.

To conclude, the suggested sequence starts with AFM tapping mode imaging because it is the least invasive technique. It may be followed by any SPM technique (i.e., SThM or SMM) in contact mode; albeit, damages are observed, but they could be managed to minimize them. High variations of *S*_11m_ signals could be expected during SMM measurements if the GO flakes could be deposited on a more conductive substrate. SEM can chemically and dimensionally modify the material. However, it is possible to limit this by pumping the chamber for a long time before imaging and acquiring larger images in order to avoid these carbon residues in a small area. Unfortunately, Raman analysis is the most invasive technique in the case of GO and this is why it must be the last analysis in this sequence, in order to carry out the correlative approach successfully.

## 4. Correlative Approach

In order to implement a correlative approach, two essential points must be mastered:

- Having the ability to image exactly the same object.
- Not damaging the object during the entire process.

The first point is resolved by the use of lithographed substrate, as described in Section 2.2.2. The potential damage has been assessed and a sequence has been determined to ensure that the most invasive techniques are performed after the least invasive techniques in Section 3.

The correlative approach, applying the sequence established in Table 1, is then performed to graphene oxide reduced at 800 °C. We should note here that the SMM will find its usefulness, as the reduction in the GO should increase the electrical conductivity of the sample.

**Figure 11:**
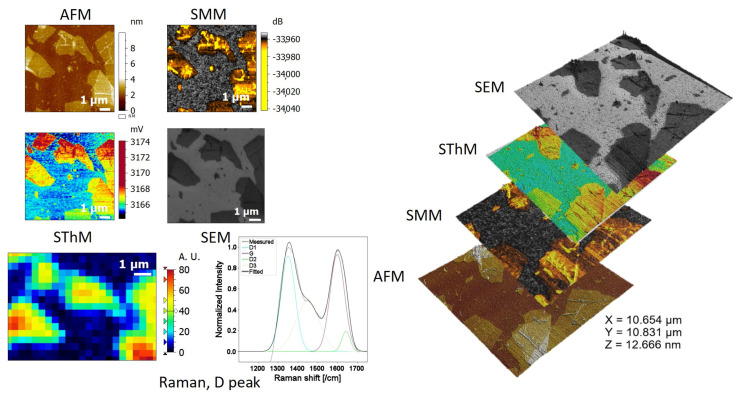
Realization of a correlative approach on reduced graphene oxide (rGO): on the left, images obtained by AFM (topography and thickness), SThM (thermal conductance), SMM (electrical mapping), SEM (morphology and lateral dimensions), and Raman spectra and mapping of the D peak for structural characterization. On the right, 3D projection and correlative overlay of the four AFM, SThM, SMM, and SEM images.

Thanks to the lithographed substrate, we were able to find and analyze the exact same flakes using AFM, SMM, SThM, SEM, and Raman spectroscopy. The results are shown in Figure 11.

The first image was produced by AFM in tapping mode and shows a group of flakes with varying numbers of layers, folds, and overlaps. The thickness measurement of GO is straightforward and can be performed either using line profiles or height histograms (Supplementary Material, S1).

In the SThM scan, we can clearly identify contrast in heat transfers between the Si substrate and rGO flakes, and even between single flake and overlapping or folded flakes. The profiles extracted (Supplementary Material, S1) from the image confirm the good agreement between the topography measurements and the SThM measurements. We observe the same value of SThM error for different single flakes. The folds identified on the RGO flakes on topography images induced peaks in the SThM profile, with an increase in the SThM error value. This could be due to a decrease in the quality of the heat transfer inside the folds, but also a decrease in the surface contact between the probe’s apex and the RGO folds.

Equally, the SMM scan corroborates the conclusions drawn from thermal analysis, regarding the visualization of the contrasts related to the morphology. Although it remains difficult to accurately estimate the electrical conductivity of reduced graphene oxide (rGO), the results obtained can nevertheless be further investigated.

The SEM images first confirm that the three previously applied SPM techniques were non-invasive and gentle on the substrate, as no flake degradation was observed. Additionally, different gray levels could be distinguished, corresponding to the number of layers present. Lateral measurements are straightforward to obtain.

Last but not least was Raman spectroscopy: by reducing laser power and exposure time and using a 50× objective instead of 100×, we obtained both interpretable mapping of D bound intensity and spectra without degrading the sample. D Peak remained present, indicating the persistence of defects in the structure. A shoulder was also observed between 1400 and 1500 cm^−1^, corresponding to the D_3_ (or D″) mode described in the literature. D″ is related to amorphous phases [62].

**Figure 12:**
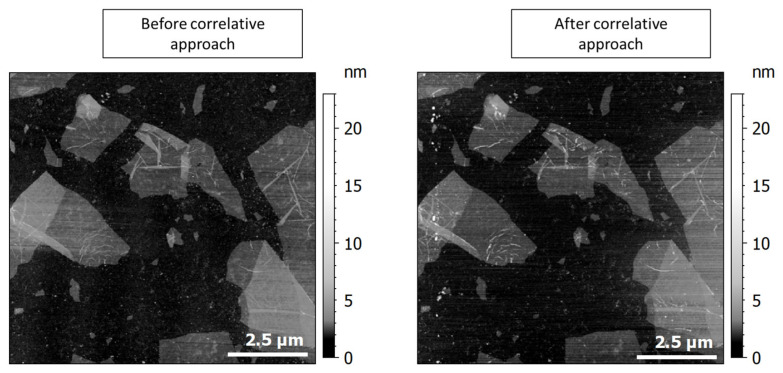
AFM tapping mode scan of the rGO flakes under investigation, before and after the whole correlative approach process.

To ensure that the whole correlative approach process on the rGO preserved the integrity of the area under investigation, an AFM control image has been produced for the area. The results are shown in Figure 12.

Figure 12 shows that after the whole process, the rGO flakes were not damaged during the process. Some contamination occurred, especially on the edges of the SPM scans: it must correspond to a scan of surface contamination when the SMM/SThM comes into contact mode. By waiting a whole night for pumping in the SEM chamber before imaging the sample, we ensured that no carbon contamination was observable. At last, the power of 7 mW with the Raman ensured that we kept the integrity of the flakes.

## 5. Conclusions

Graphene oxide is of major interest for the production of graphene-like material at a lower cost (after reduction). In order to normalize and harmonize its production, it is necessary to improve the means of characterization of such a nanomaterial. Correlative metrology is proposed as a method to obtain a comprehensive understanding of this nanomaterial and monitor its physical properties during the reduction to graphene. Though many studies agree with the need to combine different techniques on the same sample, this work provides a comprehensive investigation of a single flake, using different techniques. Indeed, inhomogeneity in properties of nanomaterials must be considered and only a series of measurements on different single flakes with the same set of techniques will help us to better understand the complexity of GO and investigate, for example, its thermal stability.

Here, we proposed tools and methods for the implementation of correlative metrology on exactly the same GO flake. First, substrates on which flakes are deposited must be selected with care, as they must meet a number of constraints. Firstly, they must be suitable for all types of techniques used. A system must be set up to allow for easy localization on the substrate. A second step deals with flake deposition. If the deposit is not optimal in terms of agglomeration and coverage rate, analysis could be very difficult. Lastly, a running sequence of the techniques has to be established before the whole process. It is important to identify which damage could be caused by each technique, and which property may be impacted.

In the work presented here, we observed that AFM in tapping mode is the least invasive of the techniques used. Then, SPM techniques in contact mode, such as SMM for electrical properties or SThM for thermal properties, were investigated. The specific probes that were employed for these kinds of modes do not always allow for a perfect control of the applied force on the sample. This could lead to the removal of the GO flake under investigation. To avoid this, we recommend a careful selection of the probe (if possible): tip apex size and cantilever stiffness. Moreover, the optimization of settings has to be performed before measuring the selected flakes. Another method of optimization is the use of an alternative AFM mode as Force–Volume (could also be called nanomechanical mode). This mode allows us to scan the surface of the sample without moving the probe or the sample in XY direction during the contact. After each contact location, the probe withdraws from the surface of the sample to move above another location before landing on it. This promising mode is already widely used for the imaging and quantification of mechanical properties, especially for soft/biological materials [63,64], avoiding the degradation of such fragile samples. This mode has also been implemented for electrical measurements [65,66] and thermal measurements [67,68] with appropriate probes and connections. This way, thermal or electrical information could be acquired in contact but without damaging the sample or the probe.

SEM and Raman could also lead to the damaging of the analyzed GO flake, not only by failing to respect the physical integrity of the flakes, but also by modifying their chemical properties: SEM by adding carbon residues and Raman spectroscopy/microscopy by possibly reducing the GO flake. Again, we recommend carefully optimizing the settings before implementation of the correlative approach. Finally, a running order could be proposed.

We have focused here on one type of material and a set of classical techniques, but it must be kept in mind that the correlative approach must be adapted for each application (material/technique), according to these three steps:

- Select a suitable substrate for all the techniques the microscopist has chosen (and to make compromises), which must also allow for easy localization of the flakes.
- Find a method to deposit a sample minimizing their agglomeration but maximizing the coverage rate
- Determine a running order for the techniques, from the less invasive to the most. The optimization of settings must be performed carefully to avoid as far as possible the damaging of the sample under investigation.

Only after all these steps may the correlative microscopy be implemented without misinterpretation, due to the modification of the sample.

These results are very promising, as they demonstrate the feasibility of monitoring the same graphene oxide flake throughout its reduction, while preserving its integrity.

## Figures and Tables

**Table 1:** Suggested running order by technique for the investigation of graphene oxide with a correlative microscopy approach.

Order of the Analysis	Technique		Targeted Properties		Possible Damage	Impact	Optimization
**1**	AFM Tapping mode		Dimensional (Height information)		-	-	
2	SMM (contact mode)	SThM (contact mode)	Electrical	Thermal	Removal of the 2D flakes	Dimensional	Lower tip force (200 nN for SMM, lower than 20 nN for SThM)
					Contamination		
3	SEM		Dimensional (XY information)		Deposit of carbon residue	Dimensional and chemical	Lower magnification, higher vacuum
4	Raman		Structural		Reduction in GO	Dimensional and chemical	Lower laser power (7 mW), less exposition time (6 s), lower magnification (50×)
					Disappearing of the 2D flakes		

## Data Availability

The original contributions presented in this study are included in the article/Supplementary Material. Further inquiries can be directed to the corresponding author(s).

## Supplementary Materials

The following supporting information can be downloaded at: https://www.mdpi.com/article/10.3390/nano15241861/s1, S1: extracted profiles (white dot line) from AFM, SMM, SThM images on exactly the same area.
