# Supplementary material for: Toward a Correlative Metrology Approach on the Same 2D Flake: Graphene Oxide Case Study—Sample Preparation and Stability Issues
Source: Nanomaterials (Basel). 2025 Dec 11;15(24):1861. doi: 10.3390/nano15241861 (PMC12735427; doi:10.3390/nano15241861)
Supplement: Supplementary file 1 [file nanomaterials-15-01861-s001.zip › nanomaterials-4028734-supplementary.pdf]

Toward a Correlative Metrology Approach on a Same 2D Flake: Graphene Oxide Case Study - Sample Preparation and Stability Issues

Supplementary material

Lydia Chibane 1,2, Alexandra Delvallée 1,*, Nolwenn Fleurence 1, Sarah Douri 1, José Morán-Meza 1, Christian Ulysse 3, François Piquemal 1, Nicolas Feltin 1 and Emmanuel Flahaut 2,*

1 Laboratoire National de Métrologie et D'Essais (LNE), 29 Avenue Roger Hennequin, 78190 Trappes, France

2 Centre Inter-Universitaire de Recherche et d'Ingénierie des Matériaux Université de Toulouse, Toulouse INP, Centre National de la Recherche Scientifique, 118 Route de Narbonne, 31062 Toulouse, France

3 Centre de Nanosciences et de Nanotechnologies, CNRS, 10 Bd Thomas Gobert, 91120 Palaiseau, France

* Correspondence: alexandra.delvallee@lne.fr (A.D.); emmanuel.flahaut@utoulouse.fr (E.F.)

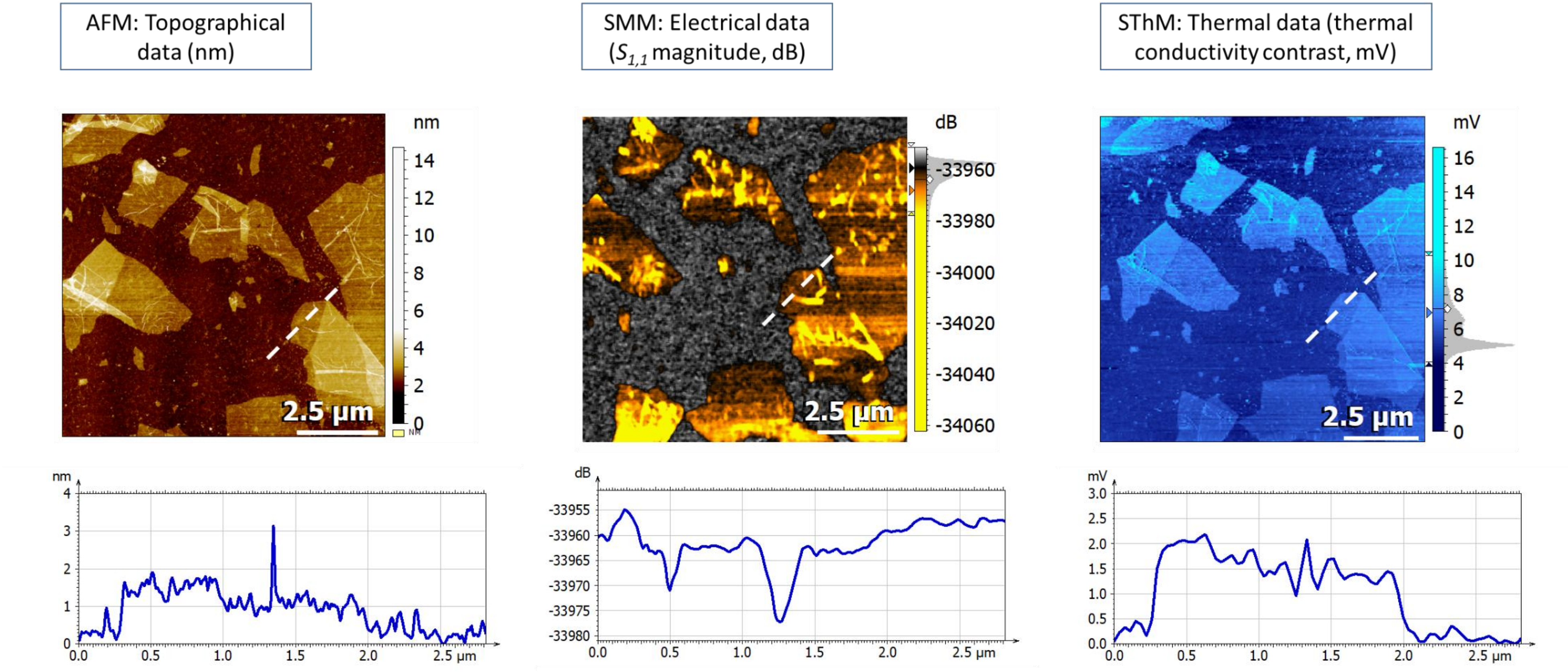

S1: extracted profiles (white dot line) from AFM, SMM, SThM images on exactly the same area.
